# Application of Fault Tree Analysis and Fuzzy Neural Networks to Fault Diagnosis in the Internet of Things (IoT) for Aquaculture

**DOI:** 10.3390/s17010153

**Published:** 2017-01-14

**Authors:** Yingyi Chen, Zhumi Zhen, Huihui Yu, Jing Xu

**Affiliations:** 1 College of Information and Electrical Engineering, China Agricultural University, Beijing 100083, China; s20153080908@cau.edu.cn (Z.Z.); yuhh1900@cau.edu.cn (H.Y.); xjing05@cau.edu.cn (J.X.); 2 Key Laboratory of Agricultural Information Acquisition Technology, Ministry of Agriculture, Beijing 100083, China; 3 Beijing Engineering and Technology Research Centre for Internet of Things in Agriculture, Beijing 100083, China

**Keywords:** Internet of Things, fault tree analysis, fuzzy neural network, fault diagnosis

## Abstract

In the Internet of Things (IoT) equipment used for aquaculture is often deployed in outdoor ponds located in remote areas. Faults occur frequently in these tough environments and the staff generally lack professional knowledge and pay a low degree of attention in these areas. Once faults happen, expert personnel must carry out maintenance outdoors. Therefore, this study presents an intelligent method for fault diagnosis based on fault tree analysis and a fuzzy neural network. In the proposed method, first, the fault tree presents a logic structure of fault symptoms and faults. Second, rules extracted from the fault trees avoid duplicate and redundancy. Third, the fuzzy neural network is applied to train the relationship mapping between fault symptoms and faults. In the aquaculture IoT, one fault can cause various fault symptoms, and one symptom can be caused by a variety of faults. Four fault relationships are obtained. Results show that one symptom-to-one fault, two symptoms-to-two faults, and two symptoms-to-one fault relationships can be rapidly diagnosed with high precision, while one symptom-to-two faults patterns perform not so well, but are still worth researching. This model implements diagnosis for most kinds of faults in the aquaculture IoT.

## 1. Introduction

The Internet of Things (IoT) has made a remarkable contributions to aquaculture production, scientific breeding, early warning and intelligent remote control. The aquaculture IoT through which we can monitor dissolved oxygen (DO) content [1], track crab behavior [2] and predict DO [3] in water has been applied in several provinces in China such as Jiangsu, Shandong and Tianjin. These applications have begun to change traditional aquaculture into intensive aquaculture [4]. However, the equipment of the aquaculture IoT is often deployed in outdoor ponds located in remote areas. Faults occurs frequently in these tough environments. Furthermore, the staff generally lacks professional knowledge and pays a low degree of attention in the remote areas. Once faults happen, expert personnel have to check the IoT on the field and carry out maintenance outdoors. This traditional fault diagnosis method causes enormous losses of resources, economic and environment [5], so effective fault diagnosis methods to guarantee the safety of the aquaculture IoT are urgently needed in this sector.

In the literature three kinds of methods have been used to diagnose faults: signal-based methods, analytical-based methods and knowledge-based methods. Rafaat proposed a signal-based method to detect faults at an early stage. The method can identify outer-race faults using stator current signals [6]. Mehra proposed an analytical-based method based on system theory and statistical decision theory for fault detection and diagnosis in linear systems [7]. As aquaculture IoT is a complex nonlinear system, the precise mathematical models needed in analytical-based methods can’t be produced in this nonlinear system, neither can the IoT provide the all process signals needed in signal-based methods. Thus a knowledge-based method was chosen to diagnose the faults in this paper, which not only can make use of fault knowledge but also can avoid the disadvantages of both signal-based methods and analytical-based methods. 

Knowledge-based methods often simulates human thoughts by using “if-then” rules to diagnose faults based on the available knowledge. These methods often use intelligent technologies like expert systems (ES), fuzzy theory, fault tree analysis and neural networks. ES have been applied to fault diagnosis in many domains [8,9]. However, ES have difficulty in knowledge acquisition and rule explosion whereby there is a trend for the systems to become larger and more complex. In order todeal with these problems, one technology is often integrated with another intelligent method in fault diagnosis. Wang integrated a neural network and a fault tree to develop an ES which made use of the advantages of the two technologies [10]. Recently, fuzzy neural networks (FNNs) and adaptive network-based fuzzy inference systems (ANFISs) have been introduced and both FNN and ANFIS algorithms have shown great promise in several field applications. However, FNN has received more attention in fault diagnosis in the recent literature. Thirty-three papers about fault diagnosis based on ANFIS, while two hundred and twelve papers about FNN could be found in the Web of Science database up to December 2016. Moreover, the two methods of hybrid fuzzy theory and neural network are different [11]. In this study, fault tree analysis is used to infer the fault rules between faults and fault symptoms, which may has function conflicts with the ANFIS. Thus we adopt FNN for the fault diagnosis in aquaculture.

In this paper an intelligent method for fault diagnosis based on a fuzzy neural network (FNN) and fault tree analysis (FTA) is proposed. In low-order linear systems, Isermann demonstrated that the robustness of the diagnosis system was enhanced by a fuzzy neural network in a continuous stirred tank reactor [12]. Li presented a method based on a hybrid of rough set theory, fault set theory, artificial neural network (ANN) and generic algorithm techniques. This method trained quicker and diagnosed at a higher level of correctness compared with a general FNN [13]. Zhang found that FNN adopts bi-directional association between fault symptoms and fault types, which was better than one-way deduction from fault symptom to fault pattern [14]. It has been proven that ANN has the strongest self-learning ability among all the knowledge-based methods [15,16], and that fuzzy set theory coupled with ANN can handle any instability caused by imprecise and inaccurate information [17], so FNN can be chosen to train relationships between faults and symptoms in aquaculture IoT.

Fault tree analysis is an inference method to show a graph of fault pairs’ relationships between faults and symptoms [18]. Chen established a system-phenomenon fault tree (SPF) to overcome the inefficiency of fault diagnosis in complex systems and identified similar faults in great number [19]. Mentes compared fuzzy FTA with the conventional method, and showed that FTA can be optimized by some other algorithms to identify system reliability or to obtain fault relationships [20]. FTA was also applied with other fault diagnosis methods to make performance better, like Bayesian-network and neural networks [21,22]. Rules in the knowledge base can be redundant in the aquaculture IoT, which results in combinatorial rule explosion, so in this study fault tree analysis and a fuzzy neural network are integrated to diagnose faults in the aquaculture IoT.

## 2. Materials and Methods

### 2.1. The Aquaculture IoT

**Figure 1:**
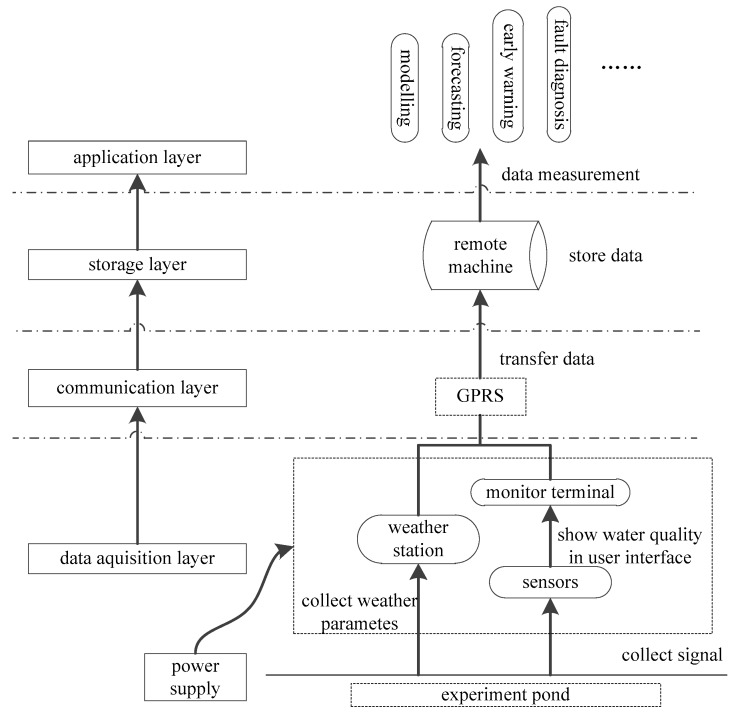
Structure of the aquaculture IoT.

The aquaculture IoT includes a data acquisition layer, transmission layer, storage layer and application layer, as shown in Figure 1. As the lowest level in the hierarchy, the data acquisition layer consists of different kinds of sensors, a weather station and monitor terminals (collectors) deployed in the experimental base. The weather station collects environment parameters in the pond microclimate. Sensor probes monitor water quality in real time by obtaining suitable signals. Then, these signals are transmitted to a monitor terminal. To make these signals readable, they are processed in a particular way, so water quality can be displayed in the user interface. Then, data is communicated via general packet radio service (GPRS, the communication layer in the hierarchy) and stored in a remote machine (storage layer). In the application layer, data are measured by different methods corresponding to different usage, like modeling, forecasting, early warning, and in this research, fault diagnosis. Besides, it’s very important to provide power, battery power and main supply, to the equipment in the base pond. In this study, the experimental base was located in Gaocheng Town, Yixing City (Jiangsu Province, China). The test ponds are 270 m long, 76 m wide, and 1.2 m deep. The IoT System consists of water quality sensors, wireless collectors, weather stations, and monitoring terminals.

### 2.2. Fault Definition in the Aquaculture IoT

Faults are complex, and each part in each layer in the overall system can fail. Because of the harsh environment, outdoor equipment can failed in the data acquisition layer or the communication layer. Sensors can be eroded by pollution and microorganisms. Wireless communications can also be disturbed or fail easily due to the environment or human errors in the complex application environment. In the storage layer and application layer, faults like software crashes occur. Power supply faults because of different power sources, like batteries, main power or photovoltaic panel power can happen. Once faults happen, it could lead to wrong decisions, waste of resources, even threaten the security of aquatic products, which would result in significant economic and human resource losses.

**Figure 2:**
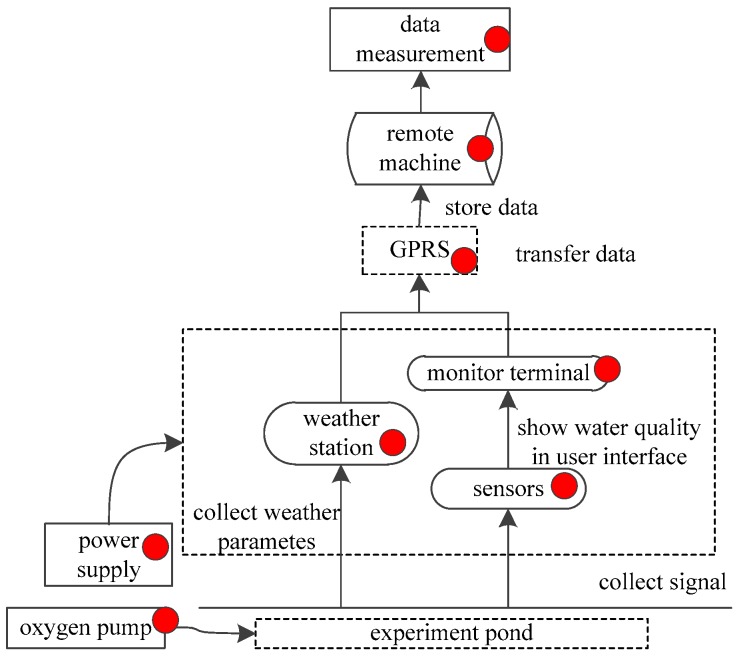
The possible locations fault occurs in the aquaculture IoT.

Thus faults definition is important and necessary for fault diagnosis in the aquaculture IoT. The faults are classified based on the fault location and numerical characteristics. In fact, a fault can occur at different locations. The red spots in Figure 2 show the possible locations where a fault can occur.

First, the faults are classified into six groups based on the fault location. Table 1 shows the six groups: sensor faults, collector faults, environmental interference, software faults, communication faults and power faults. 

The hardware faults often refers to node faults, which means the loss of part or all the specified function of a node. Sensor, collector and power supplies belong to the hardware in the IoT:

(a) The sensor faults include damaged sensor probes, sensors sinking to the bottom of the ponds, contaminated sensor probes and other sensor faults. The symptoms include high dissolved oxygen values, sensors far away from aquatic plants, dissolved oxygen values changing linearly, low dissolved oxygen values, high temperatures, abnormal rates of change in values, values below the normal threshold and higher than threshold values.
(b) The collector fault symptoms include zero readings, non-“-” reading distortion and abnormal rates of change in values.
(c) The power supply faults include battery failures, mains failures and photovoltaic panel faults. The symptoms include devices going offline and unstable voltages.
(d) The communication faults occur when data transmission is interrupted by landforms because of the narrow bandwidth of the transmission channels. The symptoms include low energy network signals, high energy network signals, weak communication signals, missing data and strong communication signals.
(e) The software faults include software errors and embedded program errors in embedded operating systems or application software, respectively. The software fault symptoms include “-” readings, unchanged data and collectors refusing to transfer logger data.
(f) Environmental interferences might affect the normal operation of the IoT, because the equipment is placed outdoors. The symptoms include missing data, zero readings and reading distortion not being “-”.

Second, the faults can be divided into six types based on their numerical characteristics. The six fault types are constant output, constant gain, constant deviation, numerical mutation, missing data, and supply voltage faults. The numerical characteristics are extracted from a deviation that the fault data differs from the normal historic data. The fault output value is represented as *f*(*t*), and the normal output value is represented as *β*_0_(*t*). The six kinds of faults are as follows:

- Constant output. Output is a constant value, and expressed by the following equation:
  (1) f(t)=C
  where C is a constant value.

**Figure 3:**
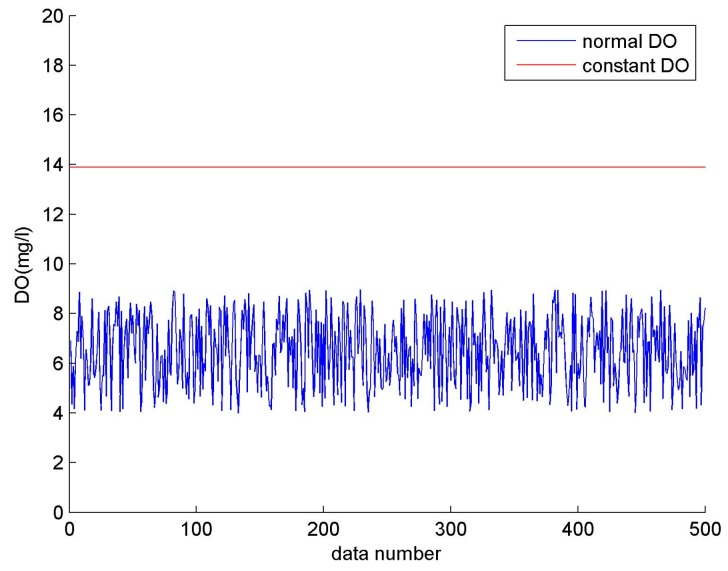
Constant output (DO).

Take the dissolved oxygen (DO) for example. A comparison between a constant failure and a normal output is shown in Figure 3. Normal DO ranges from 4–9 mg/L (blue line), while the collected value remains unchanged at 13 mg/L, and this faulty constant output is represented by the red line.

- Constant gain. Constant gain occurs when the data maintains an unchanged multiple of the normal data, expressed by the following equation:
  (2) f(t)=kβ(t)
  where *k* denotes the gain coefficient.

**Figure 4:**
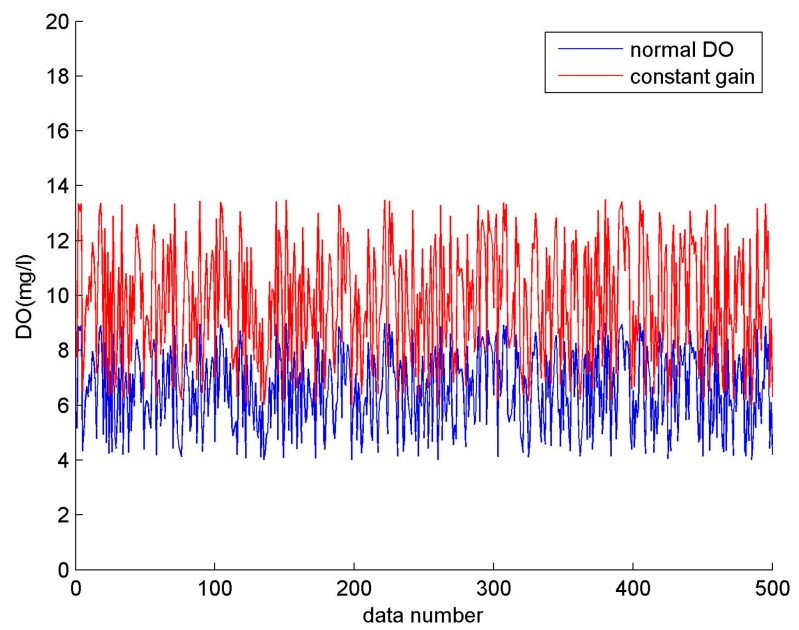
Constant gain (DO).

Figure 4 shows that there is a similar variation between the fault data represented by the red line and the normal data represented by the blue line, where b = 1.5.

**Figure 5:**
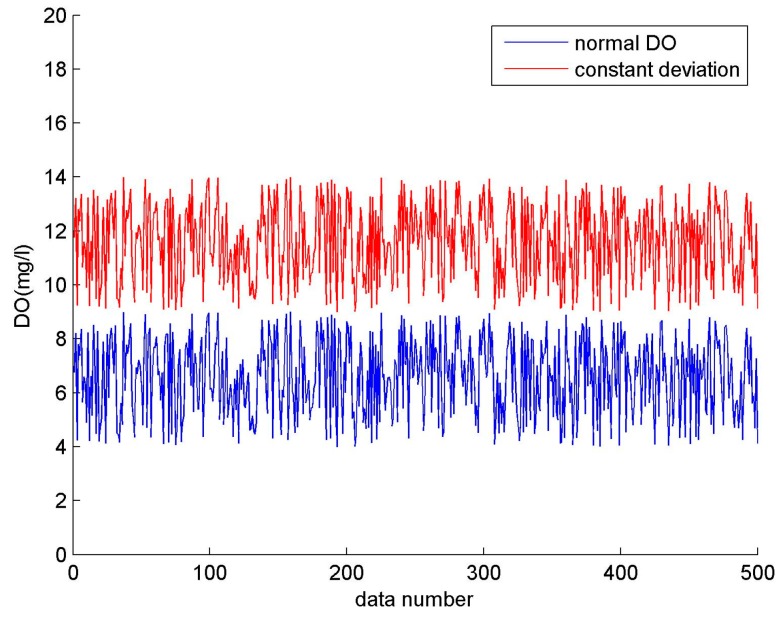
Constant deviation (DO).

**Figure 6:**
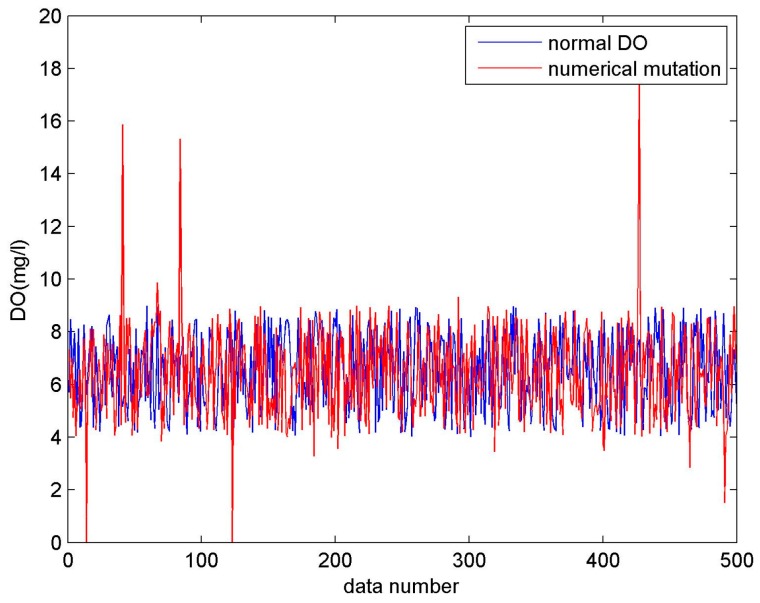
Numerical mutation (DO).

**Figure 7:**
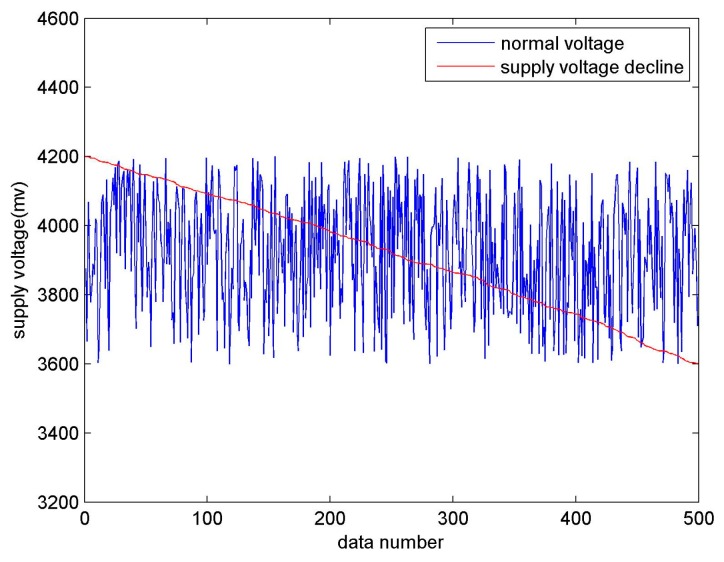
Supply voltage fault.

- Constant deviation. The collected data shows a constant bias when the sensor generates a slow drift for a long time. This fault can be expressed by the equation:
  (3) $$f(t)=\beta_{0}(t)+\Delta S$$
  where *ΔS* denotes the deviation value. As shown in Figure 5, the fault data represented by the red line and the sample data represented by the blue line differ by a constant, where *ΔS* = 5.
- Numerical mutation. The acquired value becomes overrun or is much less than normal. The fault can be expressed by the following equation:
  (4) $$f(t)=\beta_{0}(t)+\partial \delta (t)$$
  where ∂ denotes the mutation value and δ(t) is a random function representing the time a numerical mutation occurs. In Figure 6, the fault sample represented by the red line is randomly generated, and there is no law at all.
- Supply voltage fault. A remarkable feature of power failures is that the supply voltage continues to decline, even down to 3600 mV. As shown in Figure 7, the voltage fault data represented by the red line continues to decrease to 3600 mV, and even less.
- Missing data. Missing data is caused by a collector refusing to transfer data or software errors.

## 3. Results

### 3.1. Fault Diagnosis in the Aquaculture IoT

#### 3.1.1. Fault Tree Analysis

A fault tree displays a logic structure of a physical system using event symbols and logic symbols. The input and the output of the network should be determined by “IF-THEN” rules before the ANN training. Furthermore, rules acquisition becomes difficult in this complex nonlinear system, which causes redundancy and combinatorial explosion of rules. Therefore, fault trees are developed first to solve these difficulties. Then the “IF-THEN” rules are extracted from the fault trees.

**Figure 8:**
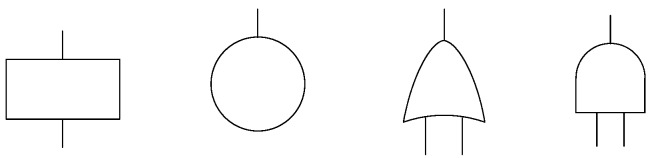
Symbols used in fault trees.

Figure 8 shows the symbols used in a fault tree. The rectangle represents an intermediate or a top event, and the circle represents a bottom event. The AND gate defines the situation that the output event will exist if all the input events exist in the same time. An OR gate presents the logical operation in the situation where one or more of the input events is required to produce the output event [23].

A fault tree is established in top-down order in this study, proceeding from faults through intermediate events to fault symptoms. The steps to establish fault trees are as follows:

Step 1: Fault-tree establishment begins with the statement of a most undesired event-fault as the top event;
Step 2: Input events need to be found, from which can be referred to higher output events;
Step 3: Repeat step 2 until the bottom events (basic events) are obtained;
Step 4: Connect all levels events with appropriate logical symbols to form the fault tree.

The rules in the qualitative knowledge base are extracted from the fault tree. The rules are expressed as:

(5) IF P, THEN Q

where *P* is a set of prerequisites connected by “AND” and “OR”, $p_{i}\in P$. *Q* is a set of conclusions, $q_{i}\in Q$. The conclusion launches when the rule meets the prerequisites.

If the logic gate is “AND”, the rule is expressed as:

(6) $$IF\;p_{1}\;AND\;p_{2},\;THEN\;q_{1}$$

If the logic gate is “OR”, the rule is expressed as:

(7) $$IF\;p_{1}\;THEN\;q_{1},\;IF\;p_{2}\;THEN\;q_{1}$$

#### 3.1.2. Fuzzy Neural Network

FNN has the advantages of both ANN and fuzzy theory. The inputs are the fault symptoms and the outputs are the faults. Parameters of the sample are chosen to describe the fault symptoms: water temperature, dissolved oxygen (DO), communication signals, etc. However, these parameters are sometimes unstable in the IoT, and cannot be expressed in a uniform way, which costs lots of memory and time in model establishment. Therefore, fuzzy set theory is used combined with a back propagation neural network (BPNN) to handle the problems.

The input vector as the fault symptom vector can be expressed as *X* = [*x_1_*, *x_2_*, …, *x_n_*]^T^. The output vector as the fault vector can be expressed as *Y* = [*y_1_*, *y_2_*, …, *y_n_*]^T^.

For the fuzzification layer, rules are converted into fuzzy sets in the form of degree of membership. Rules can be divided into numerical rules and non-numerical rules. The numerical fault symptoms are converted into three fuzzy sets for the input: ‘increase’, ‘steady’ and ‘decrease’ corresponding to the membership degree {*f1*, *f2*, *f3*}. The non-numerical fault symptoms are converted into two fuzzy sets: ‘exists’ and ‘does not exist’ corresponding to the membership degrees {*f4*, *f5*}. The values of *f1*~*f5* all range from 0 to 1, which are based on experimental and historical data of each fault symptom. 

For the output layer, the conversion is similar to the fuzzfication of the input layer. The fault situation can be expressed by five fuzzy sets: ‘exists’, ‘may exist’, ‘not sure exist or not exist’, ‘not likely to exist’ and ‘does not exist’, corresponding to specific membership degrees {*d1*, *d2*, *d3*, *d4*, *d5*}. Values of *d1*~*d5* all range from 0 to 1, which are based on experimental and historical data of each fault.

### 3.2. Application of Fault Diagnosis Method to the Aquaculture IoT

#### 3.2.1. Fault Diagnosis Using Fault Tree Analysis

**Figure 9:**
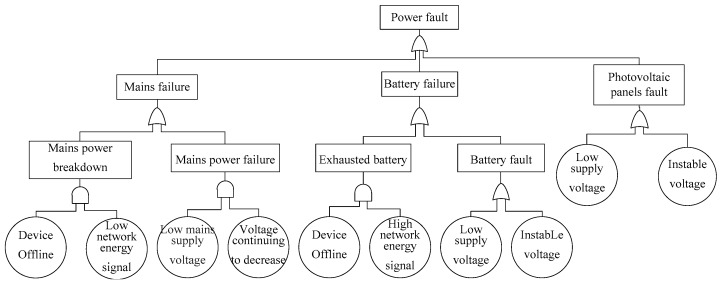
Power fault tree.

The fault tree of the IoT is divided into six sub-modules based on fault definitions: power fault tree, collector fault tree, sensor fault tree, software fault tree, environmental interference fault tree and communication modules fault tree. Figure 9 shows the power fault tree. Power faults are top events caused by mains failures, battery failures and photovoltaic panel failures which are connected by a logical “OR”:

- Main failures can be inferred from main power breakdowns or mains power failures in the main failure subtree. Main power breakdowns can be inferred from the basic symptom device offline and low network energy. Main power failures can be inferred from the basic symptoms low mains supply voltage and voltage continuing to decrease.
- Battery failures can be inferred from exhausted batteries or battery faults in the battery failure subtree. Exhausted batteries can be inferred from the basic symptoms device offline and high network energy signals. Battery faults can be inferred from the basic symptom low supply voltage or voltage instability.
- Photovoltaic panel faults can be inferred from the basic symptoms low supply voltage or instable voltage in the photovoltaic panels fault subtree.

**Figure 10:**
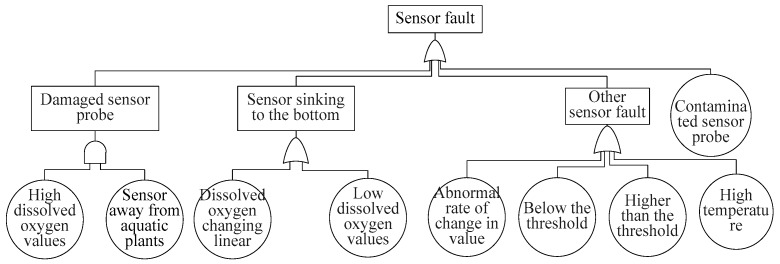
Sensor fault tree.

Figure 10 shows the sensor fault tree. Sensor faults are top events caused by damaged sensor probes, sensors sinking to the bottom of a pond, other sensor fault symptoms or the basic symptom contaminated sensor probe.

- Damaged sensor probes can be inferred from the basic symptoms high dissolved oxygen values and sensor away from aquatic plants.
- Sensor sinking to the bottom can be inferred from the basic symptom dissolved oxygen value changing linearly or low dissolved oxygen values.
- Other sensor fault symptoms can be inferred from the basic symptoms abnormal rate of change in value, below the threshold, higher than the threshold or high temperature.

Similar to the power fault and the sensor fault, communication faults are top events in the communication fault tree, caused by SIM cards, other communication faults, or missing basic symptom data.

- Other communication faults can be inferred from the basic symptoms device offline, high network energy signals and weak communicational signal.
- SIM card failures can be inferred from the basic symptoms device offline, high network energy signals and strong communicational signal.

Environmental interference can be inferred from the basic symptom data missing, reading “0” or reading distortion non “-”. Software faults can be inferred from the basic symptom reading “-”, collector refusing to transfer data from a logger or unchanged data. Collector faults can be inferred from the basic symptoms reading 0, reading distortion non “-” and abnormal rates of change in a value.

#### 3.2.2. Fault Diagnosis by Fuzzy Neural Network

The FNN includes input layer, fuzzification layer, hidden layer and output layer. In the input layer, the input vector *X* = [*x_1_*, *x_2_*, …, *x_n_*]^T^, where *n* equals 22, is the fault symptom. The input layer includes 22 nodes corresponding to the fault symptoms *X*1~*X*22. These fault symptoms are listed in Table 2.

In the fuzzification layer, the quantitative value is converted from the fault symptoms. The numerical fault symptoms, such as *X*1, *X*2, *X*3, *X*4, *X*8, *X*9, *X*10, *X*12, *X*16, *X*17, are converted into three fuzzy sets: “increase”, “steady” and “decrease”. The fuzzy sets are then represented by degree of membership, which depends on expert experience. Table 3 shows the distribution of the fault degree of membership. 

Take the degree of membership of DO for example. The value of the target DO sensor is compared with the value of other DO sensors in the same pond. All of the readings is still within the normal threshold in the same environment. Considering that change of DO content is in a certain scope in a pond, differences of DO ranging from 4 mg/L to 9 mg/L are normal, otherwise they are abnormal, so when the reading of the target DO sensor is 4 mg/L lower than the average of others, the membership degree is 0.1. When the difference is between 4 mg/L and 9 mg/L, the membership degree is 0.5. When the target reading is 9 mg/L higher than the average reading, the membership degree is 0.9.

The non-numerical fault symptoms, such as *X*5, *X*6, *X*7, *X*8, *X*11, *X*13, *X*14, *X*15, *X*18, *X*19, *X*20, *X*21, *X*22, are converted into two fuzzy sets: “exists”, “does not exist”. “Exists” and “does not exist” are expressed by the numbers “1” and “0”, respectively. When the value equals 1, a fault symptom exists, and when value equals 0, a fault symptom does not exist.

The “IF-THEN” rules are extracted from the fault tree after fuzzification, such as:

If X1 = 0.9 and X14 = 1, then Y6 = 1.
If X15 = 1, then Y7 = 1.
If X5 = 1 and X3 = 0.9 and X4 = 0.8, then Y10 = 1.
If X18 =1, then Y11 =1.

The prerequisite is the fault symptom and the conclusion is the fault. The inputs of the hidden layer are the memberships converted from the fault symptoms. The hidden layer includes 10 nodes. The Tansig function is the transfer function.

In the output layer, the output vector *Y* = [*y_1_*, *y_2_*, …, *y_n_*]^T^, where *n* = 13, is the faults. The output layer includes 13 nodes corresponding to the faults *Y*1~*Y*13. These fault symptoms are listed in Table 4.

The outputs of the output layer indicate the fault status. Output values ranging from 0–0.2 indicate the fault does not exist. Output values ranging from 0.2–0.4 indicate the fault is not likely to exist. Output values ranging from 0.4–0.5 indicate the fault is not sure to exist. Output values ranging from 0.5–0.7 indicate the fault may exist. Output values ranging from 0.7–1 indicate the fault exists.

Due to the complexity of the aquaculture IoT, one fault can cause various fault symptoms and one symptom can be caused by a variety of faults. Four types of fault patterns are chosen to test the fuzzy neural network using simulated data, such as one-to-one, one-to-many, many-to-many and many-to-one mapping relationships.

## 4. Discussion

**Figure 11:**
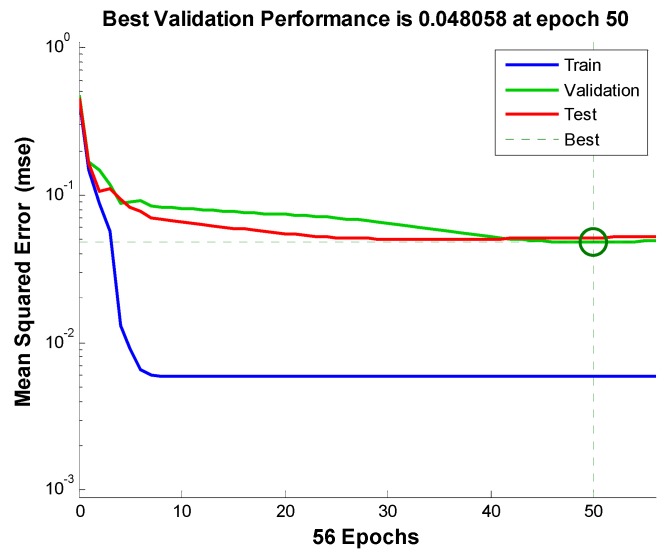
Neural network training error curve.

The FNN is established using the Matlab r2013b back propagation neural network toolbox. The input layer includes 22 nodes corresponding to the fault symptoms *X*1~*X*22. The output layer includes 13 nodes corresponding to the faults *Y*1~*Y*13. According the analysis of the 22 fault symptoms as the input layer nodes and the 13 output layer nodes for the Internet of Things (IoT) in aquaculture, we selected 10 nodes as hidden layer nodes. In the Discussion section, the results show the 10 hidden layer nodes can satisfy the fault diagnosis for the Internet of Things (IoT) in aquaculture. Training samples are obtained from practical maintenance experience. Figure 11 shows the training results of the FNN. The network converges quickly and achieves the best accuracy in epoch 50.

Table 5 shows the test results of the two symptoms (*X*3, *X*5) to one fault (*Y*1) relationship, which means two symptoms caused by one fault. The fault symptoms low network energy signal (*X*3) and device offline (*X*5) exist in this situation, which are the inputs. After the tests, the output value of mains power breakdown (*Y*1) is 1, while the rest of the outputs are much less than 0.001. Power breakdown exists based on the membership function. The results are close to the reality, so this pattern is reliable. Table 6 shows the test results of a two symptoms (*X*2, *X*18) to two faults (*Y*8, *Y*9) relationship, which means two symptoms caused by two faults. The fault symptoms high temperature (*X*2) and data missing (*X*18) exist in this situation, which are the inputs. After the test, the output value of the sensor fault (*Y*8) is 0.861683, and the communication fault (*Y*9) is 0.99811, while the rest of the outputs are much less than 0.001. The faults exist based on the membership function. The results are close to the reality, so this pattern is reliable. Table 7 shows the test results of a one symptom (*X*20) to one fault (*Y*12) relationship, which means one symptom caused by one fault. The fault symptom reading “-” (*X*20) exists in this situation, which is the input. After the test, the output value of the software fault (*Y*12) is 1, while the rest of the outputs are much less than 0.001. A software fault exists based on the membership function. The results are close to the reality, so this pattern is reliable. Table 8 shows the test results of a one symptom (*X*19) to two faults (*Y*11, *Y*13) relationship, which means one symptom caused by two faults. The fault symptom reading 0 (*X*19) exists in this situation, which is the input. After the test, the output value of the environmental interference (*Y*11) is 0.37441; the output value of the collector fault (*Y*13) is 0.31786. The faults environmental interference and collector are both not likely to exist, while both faults should exist, so this pattern has some errors when diagnosing faults, however it’s still worth researching, as the rest of the outputs are much less than 0.001.

In four relationships, the one to one, two to two, and two to one relationships perform better than the one to two relationship in the fuzzy neural network training. The one to many relationship is still worth researching. “One symptom to two faults” means that just one symptom is observed when two faults occur in the same time. This situation hardly occurs in reality, especially when a system just runs for couple of years, so that lack of data is the main reason for the worse performance.

## 5. Conclusions

This study present an intelligent method based on fault tree analysis and a fuzzy neural network to diagnose faults in the IoT of aquaculture. In this method, six fault trees are developed based on fault definition. These trees display clear logic relationships of symptoms and faults in the IoT. It also provides a basis for the fuzzy neural network modeling. The FNN model is trained to modify connection weights and thresholds, and obtain the nonlinear mapping relationships between fault pairs. The results obtained from experimental applications made on the aquaculture IoT show that this model implements diagnosis for most kinds of faults. By this study, the model provides users with fault information and maintenance suggestions. Furthermore, the application has reduced the reliance on experts, changed the traditional way of fault diagnosis, and guaranteed the safety of the aquaculture IoT.

Further work needs to be performed. First, as this aquaculture IoT has just been developed for a short time, the fault data in the aquaculture IoT is not sufficient. Thus, gathering more fault information and accumulating more experience are necessary to improve the knowledge base. Second, the one symptom to two fault symptoms relationship needs to be researched to make this method suitable for most situations. Data accumulation can improve the performance in some degree, and some algorithms may optimize the fuzzy neural network in future. Third, the choice of membership degree still relies on expert experience in this method. A proper fuzzy function should be considered to improve the model’s accuracy in future work.

## Figures and Tables

**Table 1:** The fault patterns in the aquaculture IoT.

Fault Module	Faults	Fault Symptoms	Maintenance Suggestions
Sensor	Sensor probe is damaged; Sensor sink to the bottom; Sensor probe contamination; Other	High dissolved oxygen values	Clean the probe; fix sensor in an appropriate position; Replace the probe; Replace sensor
		Sensor away from aquatic plants	
		Dissolved oxygen numerical linear	
		Low dissolved oxygen values	
		High temperature	
		Abnormal rate of change in value	
		Below the threshold	
		Higher than the threshold value	
Collector	Collector fault	Read zero	System reboot; Replace collector
		Reading distortion not “-”	
		Abnormal rate of change in value	
Power module	Low battery; Battery failure; mains failure; photovoltaic panels fault;	Device Offline Voltage instability	Restore electricity; enable additional power supply; battery replacement
Communication module	Ambient noise; communications line failure; SIM card failure	Low supply voltage	Exclude environmental interference factors; replace the SIM card; Maintenance communications line
		low mains voltage	
		Low network energy signals	
		High network energy signals	
		Weak communication signal	
		Data missing	
		Strong communication signal	
Software	Software Error; Embedded program error	Reading “-”	System reboot; Software debug
		Data unchanged	
		Refuse to transfer data logger	
Environmental interference	Environmental interference	Data missing	Eliminate interference source
		reading zero	
		Reading distortion not“-”	

**Table 2:** The inputs of the fuzzy neural network.

Input	Fault Symptom	Input	Fault Symptom
*X*1	Dissolved oxygen	*X*12	Solar power voltage
*X*2	Water temperature	*X*13	First derivative of solar power voltage
*X*3	Network energy signal	*X*14	Sensor is near the aquatic plants
*X*4	Communication signal	*X*15	DO linearly
*X*5	Equipment offline	*X*16	First derivative of water quality
*X*6	Mains voltage first derivative	*X*17	Water quality overload
*X*7	Read “-”	*X*18	Data missing
*X*8	Main voltage	*X*19	Reading 0
*X*9	RMS of mains voltage	*X*20	Reading distortion non “-“
*X*10	Battery voltage	*X*21	Collector refused to transfer data
*X*11	First derivative of battery voltage	*X*22	Data unchanged

**Table 3:** The distribution of the fault degree of membership.

Inputs	Fault Symptoms	Decrease	Steady	Increase
*X*1	DO	0.1	0.5	0.9
*X*2	Water temperature	0.2	0.5	0.8
*X*3	Communication signal intensity	0.2	0.5	0.8
*X*4	Network energy signal	0.1	0.5	0.9
*X*8	Main voltage	0.1	0.5	0.9
*X*9	Supply voltage	0.1	0.5	0.9
*X*10	Battery voltage	0.1	0.5	0.9
*X*12	Solar power voltage	0.1	0.5	0.9
*X*16	First derivative of water quality	0.1	0.5	0.9
*X*17	Water quality overload	0.2	0.5	0.8

**Table 4:** The outputs of the fuzzy neural network.

Output	Fault	Output	Fault
*Y*1	Mains power breakdown	*Y*8	Other sensor fault
*Y*2	Mains power failure	*Y*9	Communication fault
*Y*3	Battery exhausted	*Y*10	SIM card fault
*Y*4	Battery failure	*Y*11	Environmental interference
*Y*5	Photovoltaic panels fault	*Y*12	Software fault
*Y*6	Sensor probe damage	*Y*13	Collector fault
*Y*7	Sensor sink to the bottom		

**Table 5:** The test results of a two symptoms (*X*3, *X*5) to one fault (*Y*1) relationship.

	*Y1*	*Y2*	*Y3*	*Y4*	*Y5*	*Y6*	*Y7*	*Y8*	*Y9*	*Y10*	*Y11*	*Y12*	*Y13*
Actual	1	3.96 × 10^−8^	1.25 × 10^−7^	8.96 × 10^−8^	7.05 × 10^−15^	1.05 × 10^−7^	1.04 × 10^−7^	8.11 × 10^−8^	1.32 × 10^−7^	7.69 × 10^−8^	9.53 × 10^−8^	2.05 × 10^−10^	2.05 × 10^−10^
Ideal	1	0	0	0	0	0	0	0	0	0	0	0	0

**Table 6:** The test results of a two symptoms (*X*2, *X*18) to two faults (*Y*8, *Y*9) relationship.

	*Y1*	*Y2*	*Y3*	*Y4*	*Y5*	*Y6*	*Y7*	*Y8*	*Y9*	*Y10*	*Y11*	*Y12*	*Y13*
Actual	2.49 × 10^−6^	3.00 × 10^−15^	3.25 × 10^−4^	1.29 × 10^−9^	1.78 × 10^−12^	1.22 × 10^−15^	1.80 × 10^−10^	8.62 × 10^−1^	0	6.62 × 10^−7^	5.55 × 10^−17^	4.33 × 10^−8^	4.33 × 10^−8^
Ideal	0	0	0	0	0	0	0	1	1	0	0	0	0

**Table 7:** The test results of a one symptom (*X*20) to one fault (*Y*12) relationship.

	*Y1*	*Y2*	*Y3*	*Y4*	*Y5*	*Y6*	*Y7*	*Y8*	*Y9*	*Y10*	*Y11*	*Y12*	*Y13*
Actual	5.66 × 10^−13^	1.34 × 10^−10^	2.83 × 10^−7^	4.16 × 10^−8^	3.58 × 10^−8^	2.19 × 10^−7^	1.32 × 10^−7^	1.25 × 10^−7^	3.21 × 10^−8^	1.31 × 10^−11^	4.72 × 10^−9^	1	6.99 × 10^−11^
Ideal	0	0	0	0	0	0	0	0	0	0	0	1	0

**Table 8:** The test results of a one symptom (*X*19) to two faults (*Y*11, *Y*13) relationship.

	*Y1*	*Y2*	*Y3*	*Y4*	*Y5*	*Y6*	*Y7*	*Y8*	*Y9*	*Y10*	*Y11*	*Y12*	*Y13*
Actual	1.70 × 10^−9^	7.59 × 10^−10^	2.75 × 10^−8^	2.38 × 10^−14^	3.21 × 10^−11^	6.31 × 10^−8^	3.07 × 10^−8^	3.97 × 10^−^^8^	3.03 × 10^−^^7^	1.05 × 10^−^^9^	3.74 × 10^−^^1^	2.13 × 10^−^^8^	3.18 × 10^−^^1^
Ideal	0	0	0	0	0	0	0	0	0	0	1	0	1
